# Cardiorespiratory dynamics measured from continuous ECG monitoring improves detection of deterioration in acute care patients: A retrospective cohort study

**DOI:** 10.1371/journal.pone.0181448

**Published:** 2017-08-03

**Authors:** Travis J. Moss, Matthew T. Clark, James Forrest Calland, Kyle B. Enfield, John D. Voss, Douglas E. Lake, J. Randall Moorman

**Affiliations:** 1 Department of Medicine, Division of Cardiovascular Medicine, University of Virginia School of Medicine, Charlottesville, Virginia, United States of America; 2 Center for Advanced Medical Analytics, University of Virginia, Charlottesville, Virginia, United States of America; 3 Advanced Medical Predictive Devices, Diagnostics, and Displays, Inc., Charlottesville, Virginia, United States of America; 4 Department of Surgery, Division of Acute Care and Trauma Surgery, University of Virginia School of Medicine, Charlottesville, Virginia, United States of America; 5 Department of Medicine, Division of Pulmonary and Critical Care, University of Virginia School of Medicine, Charlottesville, Virginia, United States of America; 6 Department of Medicine, University of Virginia, Charlottesville, Virginia, United States of America; 7 Department of Statistics, University of Virginia, Charlottesville, Virginia, United States of America; 8 Department of Biomedical Engineering, University of Virginia, Charlottesville, Virginia, United States of America; 9 Department of Molecular Physiology, University of Virginia, Charlottesville, Virginia, United States of America; Azienda Ospedaliero Universitaria Careggi, ITALY

## Abstract

**Background:**

Charted vital signs and laboratory results represent intermittent samples of a patient’s dynamic physiologic state and have been used to calculate early warning scores to identify patients at risk of clinical deterioration. We hypothesized that the addition of cardiorespiratory dynamics measured from continuous electrocardiography (ECG) monitoring to intermittently sampled data improves the predictive validity of models trained to detect clinical deterioration prior to intensive care unit (ICU) transfer or unanticipated death.

**Methods and findings:**

We analyzed 63 patient-years of ECG data from 8,105 acute care patient admissions at a tertiary care academic medical center. We developed models to predict deterioration resulting in ICU transfer or unanticipated death within the next 24 hours using either vital signs, laboratory results, or cardiorespiratory dynamics from continuous ECG monitoring and also evaluated models using all available data sources. We calculated the predictive validity (C-statistic), the net reclassification improvement, and the probability of achieving the difference in likelihood ratio χ^2^ for the additional degrees of freedom. The primary outcome occurred 755 times in 586 admissions (7%). We analyzed 395 clinical deteriorations with continuous ECG data in the 24 hours prior to an event. Using only continuous ECG measures resulted in a C-statistic of 0.65, similar to models using only laboratory results and vital signs (0.63 and 0.69 respectively). Addition of continuous ECG measures to models using conventional measurements improved the C-statistic by 0.01 and 0.07; a model integrating all data sources had a C-statistic of 0.73 with categorical net reclassification improvement of 0.09 for a change of 1 decile in risk. The difference in likelihood ratio χ^2^ between integrated models with and without cardiorespiratory dynamics was 2158 (p value: <0.001).

**Conclusions:**

Cardiorespiratory dynamics from continuous ECG monitoring detect clinical deterioration in acute care patients and improve performance of conventional models that use only laboratory results and vital signs.

## Introduction

When we admit acutely ill patients to the hospital, we make diagnoses and initiate therapies with an aim to restore or improve health. We do not intend for patients to deteriorate and thereby require transfer to an intensive care unit (ICU). Despite our intentions, escalation of care requiring transfer to an ICU occurs at a rate of 4 to 5 for every 100 acute care admissions.[1,2] Since mortality increases with every hour of delay in transferring critically ill patients to the ICU[3,4] the acute care community has focused on identifying patients at risk of potentially catastrophic conditions by developing and implementing Early Warning Scores (EWS) from data contained in electronic medical records (EMR).[1,2,5–9] These EWS come in a variety of flavors, yet each comprises the same ingredients—intermittently measured vital signs (VS) and laboratory results (LABS) stored in the EMR.

A fundamental feature of current EWS is that they are updated only as new laboratory test results or vital sign measurements become available–this intermittent sampling may miss diagnostic patterns that would be revealed by sampling more often. Continuous physiological monitoring is often available as electrocardiographic (ECG) telemetry, but in current practice is only transiently displayed and then discarded. Analysis of continuous ECG reveals not only heart rates (HR) and dynamics such as variability, but can also extract respiratory rates (RR).[10,11] Thus, continuous ECG offers a window into cardiorespiratory dynamics, long known to hold information about the physiological status of the patient.

Here, we tested the hypothesis that the addition of cardiorespiratory dynamics measured from continuous ECG monitoring to conventional, intermittently sampled, data improves the predictive validity of models trained to detect clinical deterioration prior to ICU transfer or unanticipated death.

## Methods

### Study population

We studied consecutive adult (age > 18 years) patients admitted to acute care beds for whom continuous ECG data was available at the University of Virginia Health System, from October 11, 2013 to September 1, 2015. The 71 beds are arranged in 3 units and are under the care of a variety of hospital services, principally Cardiovascular Medicine and Cardiothoracic Surgery. An institutional electronic data warehouse archived the EMR including admission, discharge, and transfer information and a log of medical emergency team (MET) activations.

### Electronic medical record vital signs and laboratory results

At 15-minute increments, we recorded the most recent charted VS measurements—temperature, HR, blood pressures, RR, oxygen saturation (SpO_2_), oxygen flow rate, Glasgow Coma Scale—and LABS—comprehensive metabolic panel, complete blood counts, coagulation studies, arterial blood gases, lactic acid, troponin I. We excluded observations occurring after “Do Not Resuscitate” (DNR) or “Do Not Intubate” (DNI) orders or after transition to comfort measures-only (CMO).

### Cardiorespiratory dynamics measured from continuous ECG monitoring

#### Heart rate dynamics and atrial fibrillation detection

We processed the continuous ECG with three QRS detection algorithms–based on wavelet, Hilbert, and length transforms–on the ECG lead with the highest signal to noise ratio. The three resulting heartbeat time series were combined to determine the probability density of each detected heartbeat. Low confidence beats were excluded from the analysis. The QRS detection scheme used here has 97.5% sensitivity and 99.5% positive predictive value on the MIT BIH arrhythmia database hosted by PhysioNet.[12,13]

We made observations every 15 minutes of the preceding 30 minutes and calculated the mean interbeat interval, the standard deviation or HR variability, and nonlinear dynamics of HR.[14–16] From these measurements, we used a previously validated algorithm to detect the presence of atrial fibrillation (AF).[17–19]

#### Electrocardiography-derived respiratory rate and respiratory sinus arrhythmia

We estimated RR from (1) the cyclic variation in ECG waveform characteristics that result from respiratory movements and, when present, from (2) the respiratory sinus arrhythmia.[20] We analyzed 60-second interbeat interval time series windows containing 20 or more heartbeats. Windows overlapped by 75%. To analyze cyclic variation in ECG waveform characteristics, we calculated the R-S amplitude[21] and the QRS area of each detected heartbeat in every lead.[12] We defined the R-S amplitude to be the range of the ECG voltage within ±80 ms of each detected beat–maximum and minimum values were based on a quadratic fit at the extreme point. We calculated the QRS area by integrating the ECG voltage in each lead within ±80 ms of each detected beat after subtracting the median value. We then estimated the mean cardiac electrical axis between each pair of QRS area time series.[10] The unevenly sampled ECG interbeat interval time series were resampled at 4 Hz. For each time series (i.e. R-S amplitude and QRS axis), we calculated power spectra using a fast Fourier transform and selected the lowest RR between 4 and 55 breaths per minute that had power of at least 95% of the maximum. As a measure of the degree of respiratory sinus arrhythmia, we recorded the amplitude of the RR peak.

We combined the multiple measures of ECG RR following the methods of Nemati, Li, Clifford and co-workers.[11,22] Briefly, each RR estimate was processed in a Kalman filter using the spectral purity to estimate the observation noise. A fused ECG RR was calculated by averaging the multiple estimates weighted by their innovation. We averaged the ECG RR and respiratory sinus arrhythmia amplitude measurements in 30-minute windows with 50% overlap.

### Outcomes

The primary composite outcome was clinical deterioration leading to any of the following events: (1) unanticipated direct transfer to the ICU, (2) MET activation with subsequent transfer to the ICU within 12 hours, (3) emergent or urgent (within 1 hour) operating room procedure with subsequent transfer to the ICU, or (4) unexpected death within 24 hours of last time recorded in a telemetered bed. In cases without unexpected death, we did not classify events with ICU dwell times of shorter than 4 hours. We defined the time of the event as the time of ICU transfer, urgent surgery, death, or MET activation that preceded death. Two investigators reviewed unexpected deaths and transfers to the ICU for clinical deterioration. A third reviewer adjudicated cases in which there was disagreement.

### Statistical analysis

We calculated the median values and interquartile ranges (IQR) for continuous variables and reported the counts and percentages of categorical measurements. During periods of continuous ECG monitoring, we developed multivariable prediction models to predict the risk of the primary composite outcome in the next 24 hours. We labeled observations during the time window prior to the event as cases, and observations outside this timeframe as controls.

In model development we adhered to the Transparent Reporting of a multivariable prediction model for Individual Prognosis Or Diagnosis (TRIPOD) statement recommendations.[23] To minimize the inclusion of redundant measurements and to avoid overfitting, we eliminated highly correlated predictors. We evaluated 48 candidate predictors derived from 39 component measurements (7 vital sign measurements, 25 lab results, 7 ECG calculations), including the slope or change of HR and ECG RR over windows of up to 24 hours. As new AF with rapid ventricular response commonly occurs and complicates critical illness, we also tested the interaction between current AF status and that of the initial rhythm detected. To impute missing values, we carried the last value forward for up to 24 hours for charted VS and for up to 48 hours for LABS; thereafter we imputed median values. We allowed our pre-specified candidate predictors to have non-monotonic and non-linear associations by modeling them with restricted cubic spline transformations.[24] We retained all significant higher-order factors. We adjusted for the repeated and correlated observations within individual patients with procedures that modify the variance-covariance matrix, and we quantified predictive accuracy using a concordance index (C-statistic).[24]

We developed a first set of models from each independent data source (i.e. VS, LABS, ECG monitoring data). We conducted a second round of model development by modeling from all the pairwise combinations of independent data sources. Finally, we developed a combined model that integrated all available data sources. At each round to quantify the impact of adding the additional data source to the preceding model, we calculated the C-statistic and a categorical NRI for a change of 1 decile in risk. To test the hypothesis that the addition of cardiorespiratory dynamics to a model comprised of charted vital signs and laboratory results improves predictive validity, we calculated the probability of achieving the difference in the likelihood ratio χ^2^ of the two models accounting for the difference in degrees of freedom between the two models and report this as a p-value. In sensitivity analysis we stratified the evaluation of model discrimination by the admitting hospital service. We also calculated several well-known EWS and compared their performance to that of our univariate and multivariate models.

We validated these models internally using bootstrap resampling (TRIPOD Type 1b model study) to estimate the performance on a new sample of observations from the same patient population, and report the bias-corrected C-statistics.[23] We justify bootstrapping methodology over split-sample or cross-validation techniques by noting that the estimates generated by these alternative strategies are less stable (different splits lead to different results) and exhibit greater bias (samples that vary only by chance will probably show similar performance).[23,25]

## Results

### Characteristics of admissions

**Fig 1:**
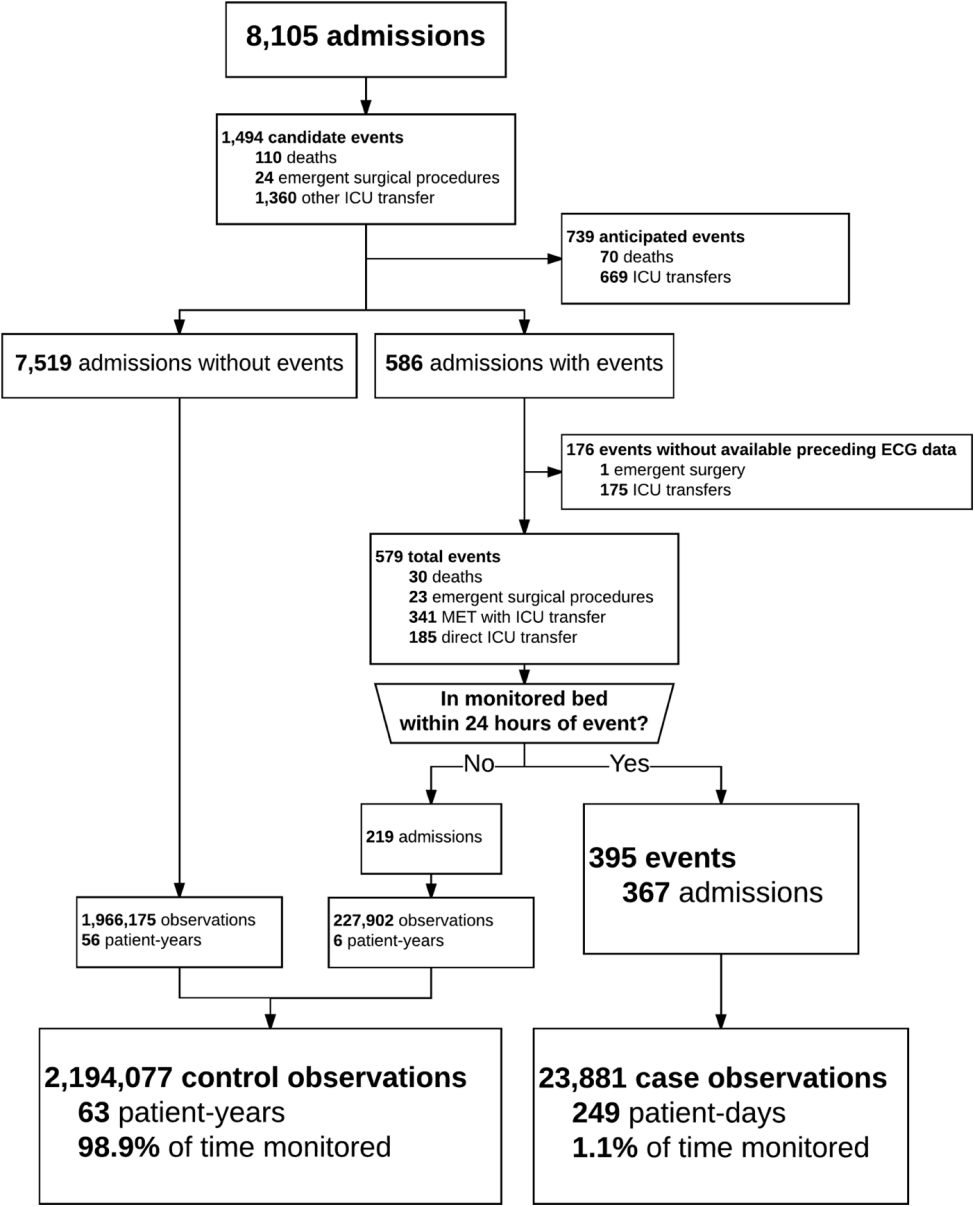
Flowsheet of analysis.

We studied 8,105 consecutive admissions with available continuous ECG data. After exclusion of observations occurring after DNR, DNI, or CMO orders, we analyzed the remaining 2.2 million observations (63.3 patient-years; **Fig 1**). **Table 1** provides descriptive statistics of the cohort. The majority of patients were admitted to either Cardiovascular Medicine or Cardiothoracic Surgery services (5,504 admissions [68%], 81% of all patient-days). The median age was 65 (IQR: 55–75; range: 18–99) years and the median hospital length of stay was 4 (2–7) days.

**Table 1:** Characteristics of admissions by primary outcome.

	No Event	Event
**Percentage (n)**	93 (7,519)	7 (586)
**Age, years**	65 (55–75)	67 (57–76)
**Male**	59 (4,457)	60 (352)
***Race***		
**White**	81 (6,077)	82 (482)
**Black**	17 (1,279)	16 (95)
**Other**	2 (163)	2 (9)
***Admitting Service***		
**Cardiology**	49 (3,711)	42 (250)
**Cardiothoracic Surgery**	19 (1,441)	17 (102)
**Other Medicine**	16 (1,194)	24 (139)
**Other Surgery**	16 (1,173)	16 (95)
***Number of Events***		
**1**	—	94 (551)
**2**	—	6 (33)
**≥ 3**	—	<1 (2)
***Event Criteria***		
**MET activation**	—	64 (371)
**Direct Transfer**	—	32 (185)
**Unanticipated Death**	—	5 (30)
**Emergent Surgery**	—	4 (23)
***Hospital Outcomes***		
**Length of Stay, days**	4 (2–7)	11 (7–19)
**ICU Length of Stay, days**	0 (0–1)	4 (2–7)
**Mortality**	0.4 (28)	17.2 (101)

Values are percentages (counts) or median value (interquartile range).

### Laboratory tests, vital signs, continuous ECG measurements

**Fig 2:**
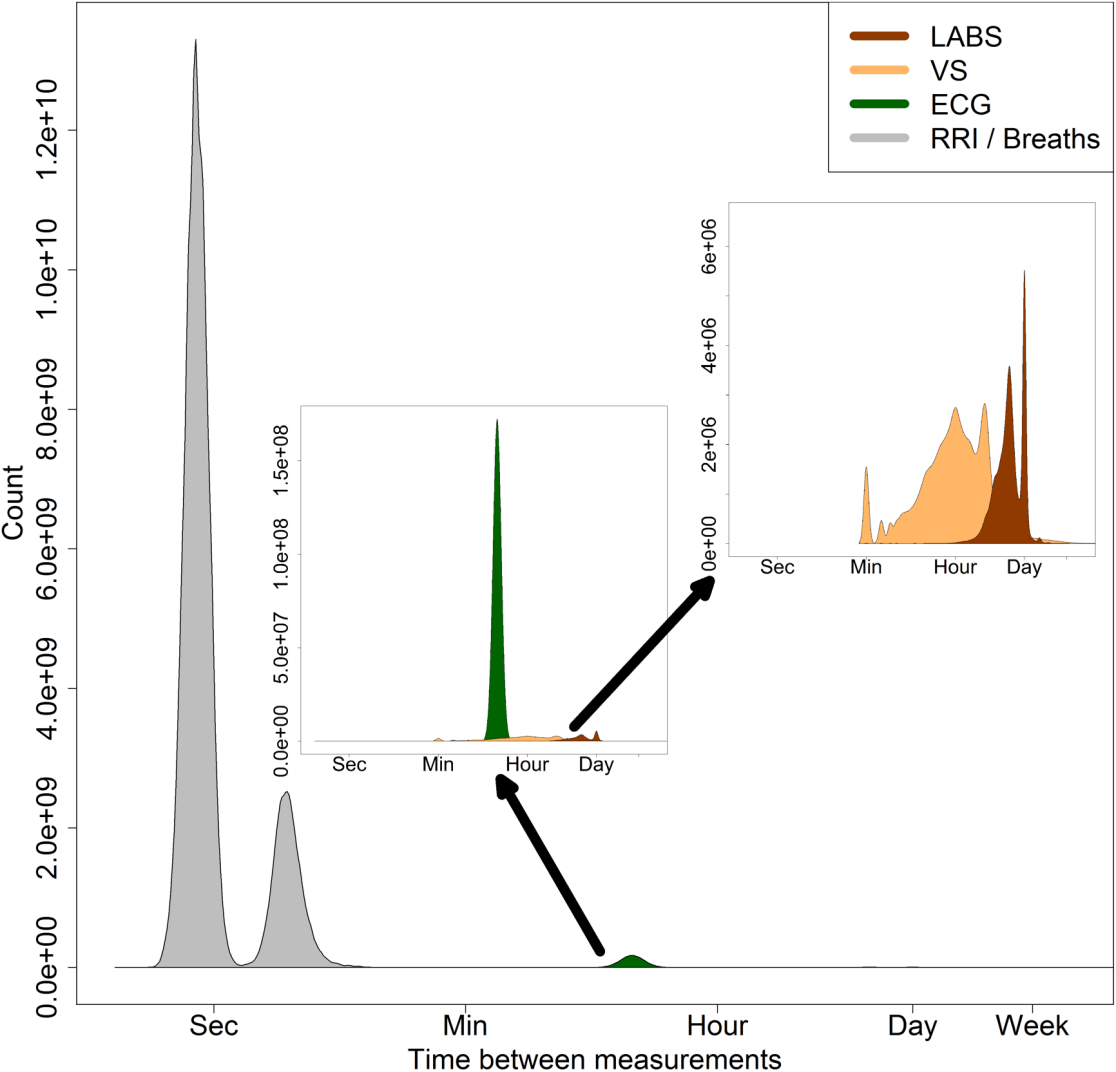
Histograms of the time between measurements, logarithmic scale. The area of each histogram indicates the quantity of measurements available. R wave to R wave (RR) inter-beat and inter-breath intervals (gray) occur on the order of seconds, 2.6 billion heart beats and 600 million breaths. (Middle Inset) Features from the continuous ECG monitors (green) were aggregated at q15 minute increments. (Upper Right Inset) Standard nursing vital signs (yellow) were most frequently obtained at every 1 to 4 hour intervals, 4.1 million measurements. Automated entries into the EMR are evident at every 1 minute. Laboratory results (red) were most frequently measured every 12 hours or daily.

**Fig 2** shows that the numbers of measurements varied widely, from 1.8M LABS (right inset, brown) to 4.1M VS (right inset, beige) to 600M breaths (left, gray) and 2600M heartbeats (left, gray). The green distribution in the middle inset represents 35M every-15-minute calculations from continuous physiologic measures used for the ECG monitoring statistical models.

**Fig 3:**
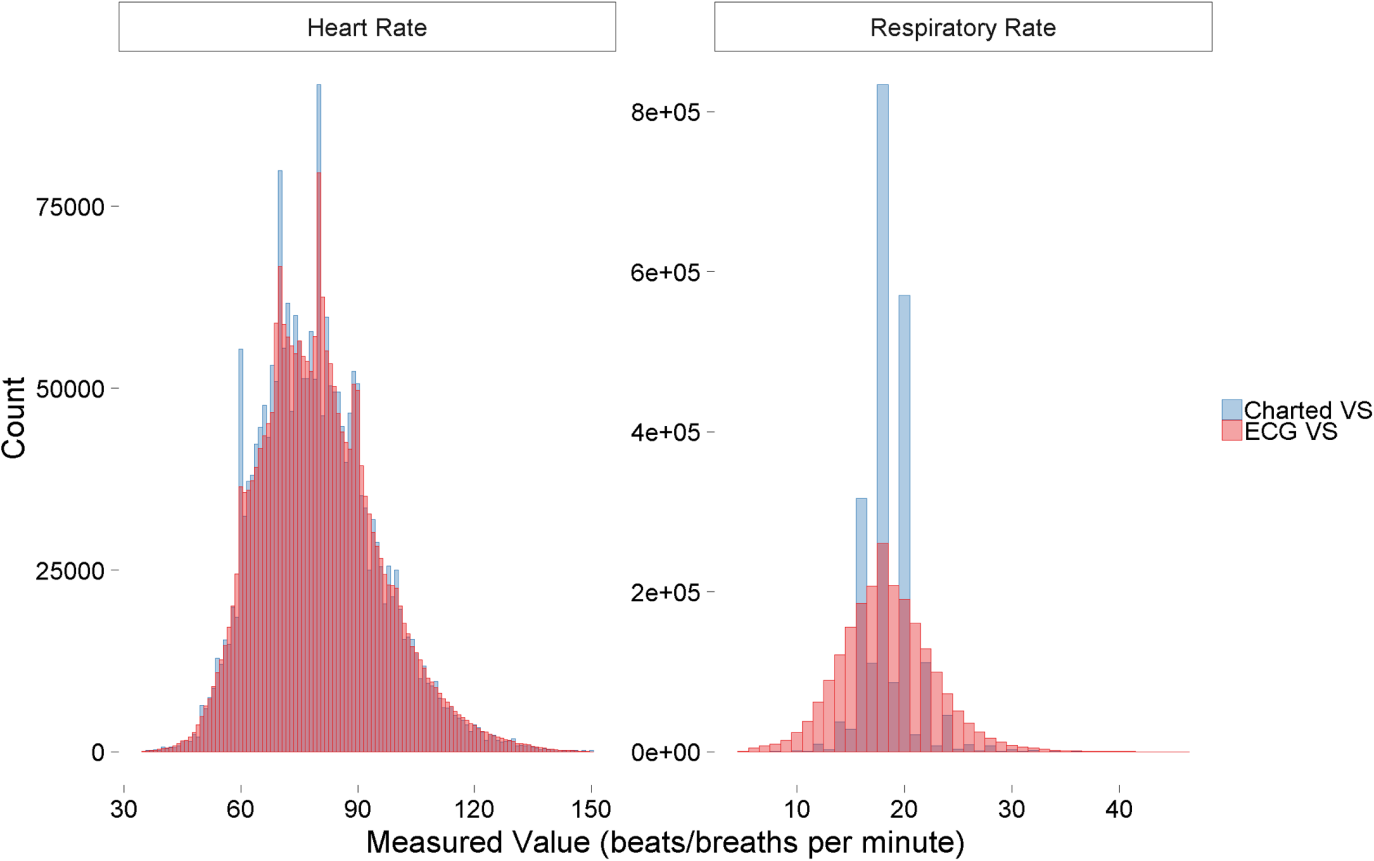
Distributions of measured heart rate and respiratory rate. Heart rate (left) and respiratory rate (right) measurement distributions according to source, charted vital signs (VS; blue) vs electrocardiography (ECG; red) where each source had equal numbers of measurements.

There was good agreement of HR and RR from the charted VS and the ECG analysis. RR from charted VS was most frequently one of three values: 18 (15.0%), 20 (11.7%), and 16 (9.9%) breaths per minute (**Fig 3**). The distribution of ECG RR, as expected, was more continuous. AF was the first detected rhythm in 655 admissions (8.1%) and was present in 9.3% of all observations during ECG monitoring.

Missing data were extremely rare among continuously measured data (range: 0–2.4%) and among most intermittent VS (0.1%) with the exception of Glasgow Coma Scale (7.2%) and oxygen flow rate (55.0%). Missing data were more or extremely common among LABS (basic metabolic panel: 14.4%, complete blood count: 17.0%, coagulation studies: 48.7–65.6%, liver function tests: 75.4%, troponin I: 86.2%, and lactic acid: 95.0%).

### Outcomes

The primary composite outcome of deterioration leading to unanticipated ICU transfer or death occurred 755 times in 586 admissions (7%). Many events triggered MET activations (n: 341; 45.2%). While 159 patients died within 24 hours of last time recorded in a telemetered bed, only a minority were unanticipated and not accompanied by new DNR, DNI, or CMO orders (n: 30; 4% of all events). Admissions with events had 3-fold longer median hospital lengths of stay (11 vs 4 respectively) and a 43-fold increase in hospital mortality (17.2% vs 0.4% respectively; **Table 1**). Continuous ECG data was available prior to 395 of these events (52%) with which we developed new statistical models.

### Models derived from individual data sources

**Fig 4:**
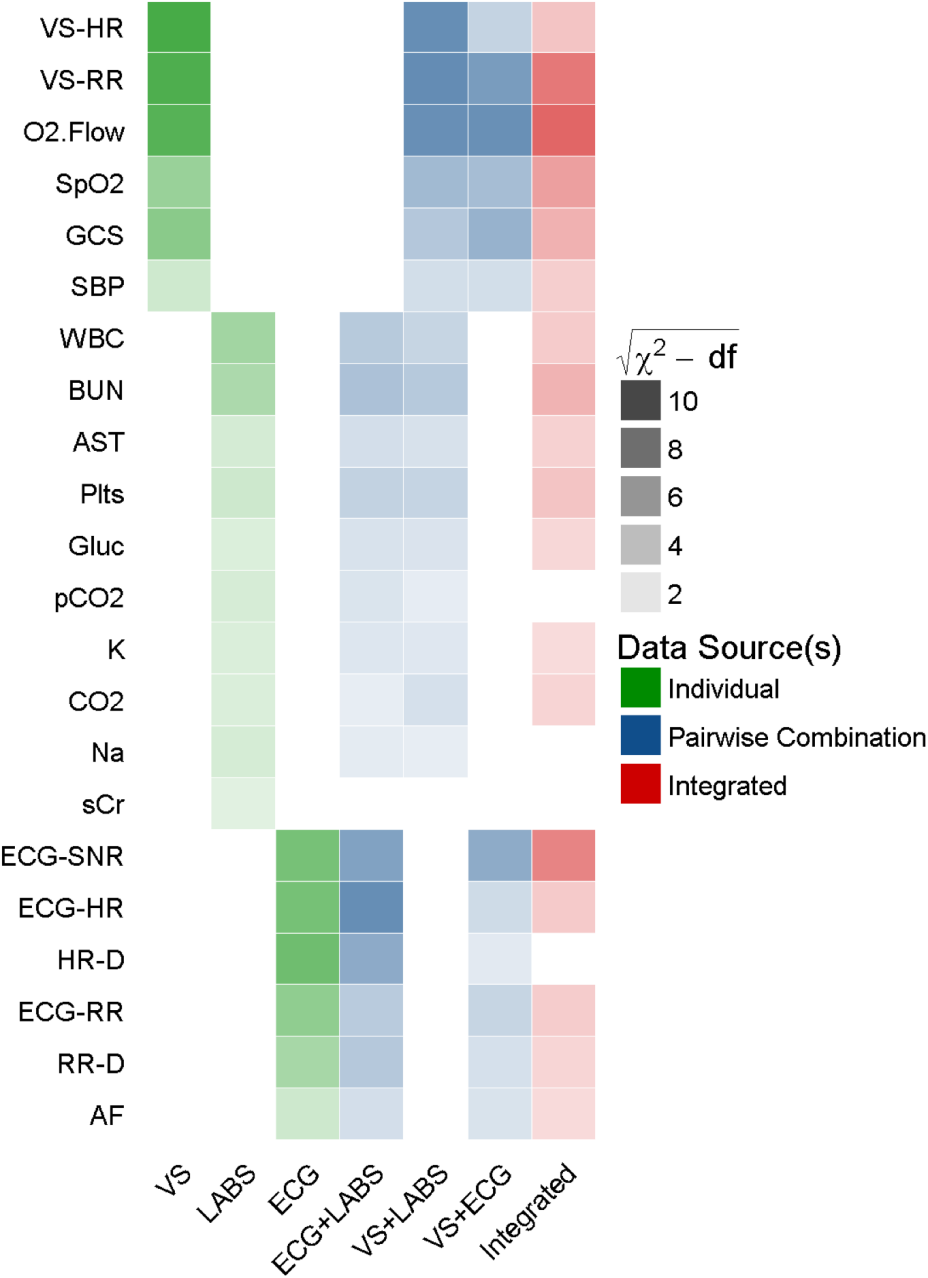
Relative statistical significance of component predictors included. Heatmap depiction of statistical significance of predictors (rows) in each model (columns) with corresponding C-statistics. Saturation or transparency depicts the statistical significance as calculated by the square root of (χ^2^ minus degrees of freedom (df)) and can be likened to the absolute value of a Z score adjusted for the degrees of freedom associated with a predictor due to higher order terms. Hue or color represents the data source: individual data sources (green), pairwise combinations (blue), fully integrated model using all available data (red). Predictors that failed to achieve statistical significance in any of the models are not displayed. VS-HR: charted heart rate, VS-RR: charted respiratory rate, O2.Flow: charted oxygen flow rate, SpO2: charted oxygen saturation, GCS: Glasgow Coma Scale, SBP: systolic blood pressure, WBC: white blood cell count, BUN: blood urea nitrogen, AST: aspartate aminotransferase, Plts: platelets, CO2: carbon dioxide, Glu: glucose, pCO2: partial pressure of carbon dioxide, K: potassium, Na: sodium, sCr: creatinine, ECG-SNR: Signal-to-Noise metric from ECG-derived respirations, ECG-HR: heart rate from continuous ECG, HR-D: trend of HR over prior 24 hours, ECG-RR: ECG-derived respiratory rate, RR-D: trend of RR over prior 24 hours, AF: atrial fibrillation, EMR: electronic medical record.

A model derived from intermittently measured VS had a bias-corrected C-statistic of 0.687 (optimism: 0.008) and all predictors but temperature were associated with clinical deterioration (**Fig 4**). A model derived from intermittently measured LABS had a C-statistic of 0.629 (optimism: 0.025). A model derived from cardiorespiratory dynamics featuring HR, ECG-RR, AF, and the slopes of both HR and ECG-RR had a C-statistic of 0.651 (optimism: 0.010).

### Models derived from integrated data

**Fig 5:**
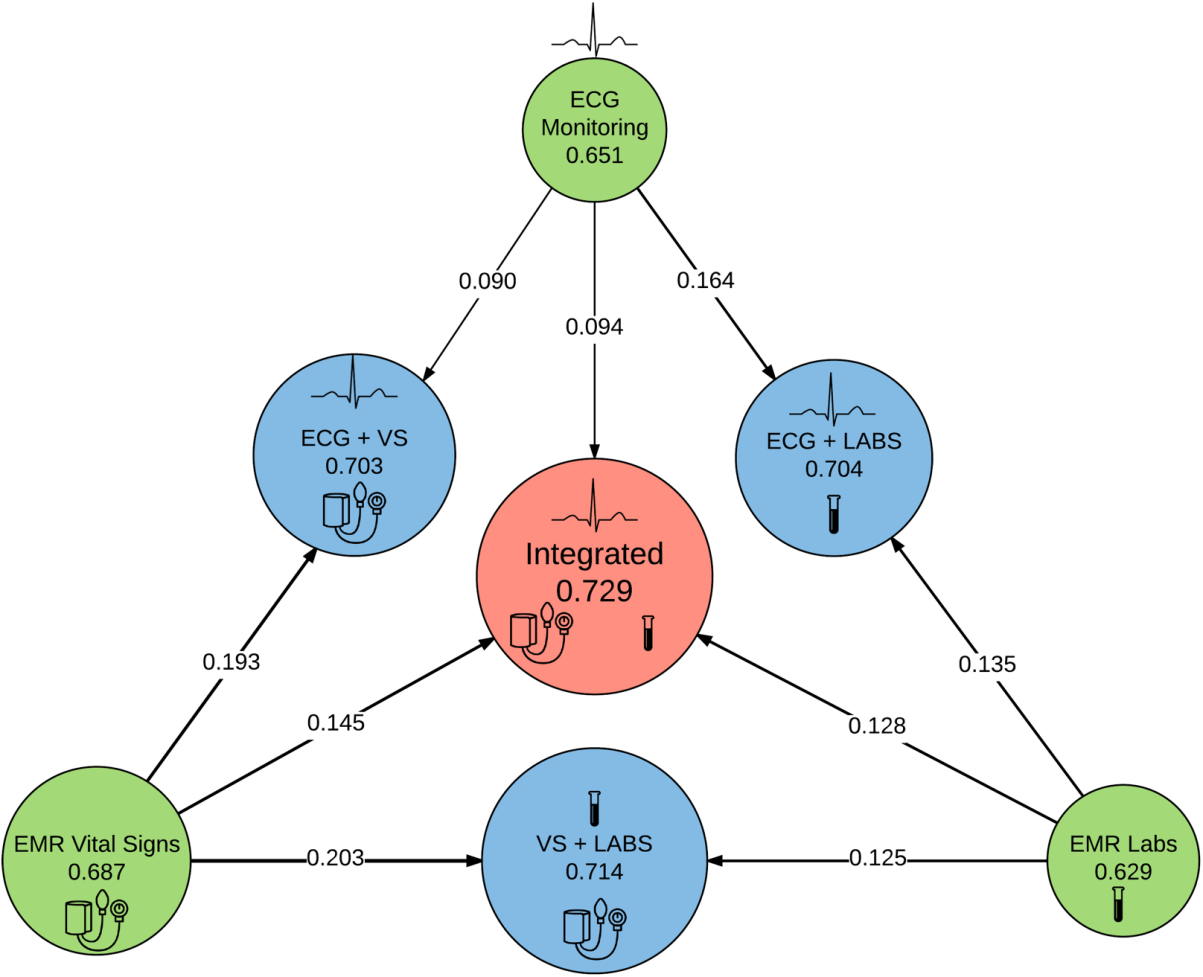
Schematic of sequential integration of additional data sources. Each circle represents an individual model with the C-statistic reported below each model abbreviation. Area of each circle is proportionate to the total likelihood ratio test χ^2^ minus two times the degrees of freedom (df) of all non-intercept terms.

Combining significant terms from the VS model to the cardiorespiratory dynamics model resulted in a C-statistic of 0.703 (optimism: 0.011) with categorical NRI of 0.09 and 0.19 for cardiorespiratory dynamics and VS respectively (**Fig 5**). Adding cardiorespiratory dynamics to the LABS model resulted in a C-statistic of 0.704 (optimism: 0.011) with NRI of 0.14 and 0.16 for LABS and cardiorespiratory dynamics respectively. Combining VS with LABS resulted in a C-statistic of 0.714 (optimism: 0.014) with NRI of 0.20 and 0.13 for VS and LABS respectively. A fully integrated model using all available data sources had the best C-statistic: 0.729 (optimism: 0.014) with NRI of 0.15, 0.13, and 0.09 for the addition of VS, LABS and cardiorespiratory dynamics respectively. The difference in likelihood ratio χ^2^ between integrated models with and without cardiorespiratory dynamics was 2158 with a difference of 13 degrees of freedom (p value: <0.001). The most important predictors were RR, supplemental oxygen use, SpO_2_, and ECG signal to noise ratio (ECG-SNR).

### Effect of admitting hospital service

The incidence of clinical deterioration ranged from 1.15 to 2.24 per 100 monitored patient-days (**Table 2**). While there were differences in model discrimination amongst various hospital services, as additional data sources were integrated into the model, and consistent with our more general analysis, the predictive validity incrementally improved. The fully integrated model had C-statistics of 0.715, 0.716, 0.775, and 0.796 for Cardiovascular Medicine, Cardiothoracic Surgery, Other Medicine, and Other Surgery respectively. The minimal anonymized data set necessary to replicate regression model analysis above is publicly available on the University of Virginia Dataverse (Moss, Travis 2017, “Replication Data for: Cardiorespiratory dynamics measured from continuous ECG monitoring improves detection of deterioration in acute care patients: A retrospective cohort study”, doi:10.18130/V3/MKY17T, University of Virginia Dataverse).

**Table 2:** Event incidence and model performance stratified by admitting service.

	Cardiology	Cardiothoracic Surgery	Other Medicine	Other Surgery
Admissions (%)	3,961 (49%)	1,543 (19%)	1,317 (16%)	1,284 (16%)
Patient Years Monitored (%)	33.1 (52%)	11.4 (18%)	9.5 (15%)	9.2 (15%)
Events (%)	207 (52%)	48 (12%)	78 (20%)	62 (16%)
Event Rate (per 100 patient-days)	1.71	1.15	2.24	1.84
***C-statistic***				
Vital Signs	0.699	0.631	0.710	0.688
Lab Results	0.627	0.684	0.674	0.664
ECG Monitoring	0.666	0.587	0.664	0.673
VS + LABS	0.705	0.710	0.760	0.758
VS + ECG	0.706	0.648	0.732	0.744
ECG + LABS	0.685	0.697	0.742	0.765
Integrated	0.715	0.716	0.775	0.796

### Comparison to other early warning scores

In evaluating all univariate predictors, our newly derived models, and common EWS, we found that the top 5 performing models for the composite outcome were those newly developed and presented in this paper with the exception that the electronic Cardiac Arrest Risk Triage (eCART) score[8] outperformed our model derived from only continuous ECG metrics, and both the National Early Warning Score (NEWS)[7] and the VitalPac Early Warning Score (ViEWS)[6] outperformed our model derived only from LABS (**Table 3**). Systemic Inflammatory Response Syndrome (SIRS)[26] came next as the 11^th^ best model evaluated while qSOFA[9] and Acute Physiology and Chronic Health Evaluation (APACHE) II[27] both performed more poorly than the univariate predictor of mean HR taken from either the continuous ECG monitor or from the EMR charted VS.

**Table 3:** Ranking of model/predictor performance for primary outcome.

Predictor or Model	Rank	C-statistic	d.f.	Χ^2^ –d.f.
Integrated	1	0.742	38	441.9
VS + LABS	2	0.726	25	378.2
ECG + LABS	3	0.713	26	285.2
VS + ECG	4	0.712	25	382.3
VS	5	0.692	16	335.7
eCART	6	0.674	15	235.8
ECG	7	0.660	19	201.6
ViEWS	8	0.652	6	193.8
NEWS	9	0.652	6	193.0
LABS	10	0.649	29	153.7
SIRS	11	0.641	3	162.3
ECG HR	12	0.627	1	93.3
MEWS	13	0.622	6	162.5
EMR HR	14	0.621	1	96.9
qSOFA	15	0.593	2	94.6
O2 Flow	16	0.589	1	100.7
ECG RR	17	0.589	1	49.2
BUN	18	0.575	1	39.2
NEWS component >3	19	0.569	1	69.9
APACHE II	20	0.568	25	-8.9

All univariate predictors, models, and Early Warning Scores were evaluated in identical fashion where observations within 24-hours of the primary outcome were labeled as cases and all other at-risk observations were labeled as controls. Ranking is from best discrimination to worst as quantified by C-statistic (non bias-corrected). We also report the degrees of freedom and the total statistical significance or χ2 penalized by added complexity by subtracting two times the degrees of freedom of the model. VS: model derived from electronic medical record vital signs; LABS: model derived from electronic medical record laboratory results; eCART: electronic Cardiac Arrest Risk Triage score; MEWS: Modified Early Warning Score; qSOFA: quick Sequential [Sepsis-related] Organ Failure Assessment; ViEWS: VitalPac Early Warning Score; NEWS: National Early Warning Score; BUN: blood urea nitrogen; NEWS component > 3: any single NEWS component score >3; APACHE II: Acute Physiology and Chronic Health Evaluation; SIRS: Systemic Inflammatory Response Syndrome; ECG: electrocardiography; HR: heart rate; d.f.: degrees of freedom.

### Example cases

**Fig 6:**
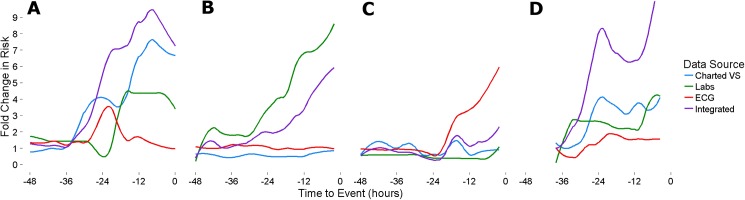
Example cases of deterioration. **A:** Patient post-procedure day #1 from stenting of left posterior tibial artery for non-healing ulcer deteriorated into mixed cardiogenic and septic shock. Here charted vital signs were sufficient to identify deterioration in the several hours preceding ICU transfer. **B:** Patient with heart failure undergoing evaluation for coronary artery bypass grafting quickly deteriorated due to acute renal failure. Progressive derangement of laboratory results identified the deterioration without appreciable change in charted vital signs or continuous ECG monitoring. **C:** Patient post-operative day #3 from left renal vein transposition who developed abdominal pain with associated tachycardia. CT imaging demonstrated intraabdominal hemorrhage and subsequent hemoglobin level had dropped from 9.6 to 6.0. EMR charted vital signs and lab results failed to appreciate a trend that was apparent for several hours by analytics of ECG monitoring. **D**: Patient admitted with acute hypoxic respiratory failure and acute kidney injury post-procedure day #0 from chest tube insertion to drain a pleural effusion. While charted vital signs and laboratory results were abnormal, integration of all available data sources accentuates her increasing risk in the several hours prior to ICU transfer.

**Fig 6** depicts 4 different admissions marked by deterioration resulting in unanticipated ICU transfer. In **panel A**, developing shock is recognized by models derived from multiple sources, but charted VS alone would have been sufficient to identify this patient several hours before ICU transfer. **Panel B** provides an example of a patient with developing confusion and acute renal failure where laboratory results but neither of the other data sources identified her trajectory. **Panel C** represents a patient on post-operative day #3 from an uncomplicated vascular surgery whose progressive abdominal pain and tachycardia prompted CT imaging and a lab draw, both of which were consistent with intra-abdominal hemorrhage leading to urgent exploratory laparotomy. ECG monitoring of cardiorespiratory dynamics identified this deterioration long before the other diagnostic tests were obtained that prompted urgent surgery. **Panel D** represents a patient with progressive respiratory failure and renal failure despite therapeutic drainage of a pleural effusion. While all data sources identified her as at increased risk, the integration of all data sources accentuated the severity and acuity of her deterioration.

## Discussion

We studied continuous physiological monitoring prior to adverse events in patients hospitalized in acute care beds. We found that cardiorespiratory dynamics measured from continuous ECG monitoring improved the predictive validity of models based on intermittent measurements of VS and LABS.

While multiple parameters helped to detect the deteriorating patient, RR, supplemental oxygen use, and SpO_2_ were the most important. These findings underscore the need for accurate counts of RR and assessment of respiratory status. The quality of the ECG signal, measured as the ECG-SNR, was also important; underscoring that careful attention to subtle changes in skin properties—like temperature, dryness, and texture—may provide warning of impending patient decline.

Detecting the deteriorating patient on the hospital ward is a major goal. The causes are varied, and range from underestimation of the admission diagnosis to the development of new and unrelated illnesses. The consequences range from altered management plans on the ward to ICU transfer and in some cases even to cardiac arrest and death. Physicians and nurses agree that early warning signs are often present, but are sometimes recognized only in retrospect. Beginning with APACHE for ICU patients in 1981[28] and EWS for acute care patients in 1997[29], many systems have been developed using charted elements–VS, level of consciousness, laboratory test results. In 2008, Smith and coworkers reviewed more than 60 such systems using single or multiple predictive parameters.[30,31] These systems benefit from systematically examining all the available data, and from the clinical wisdom of their developers. They lack, however, information from continuous physiological monitoring that is available for many hospitalized patients.

We hypothesized that the cardiorespiratory dynamics held information about the changing state of the hospital patient vulnerable to deterioration leading to ICU transfer, and we tested this hypothesis using advanced ECG signal processing techniques and a large, well-annotated database. From the ECG waveform signals, we determined linear and non-linear dynamical properties, identified AF, and detected respiration. From individual review of charts, we selected only patients whose ICU transfer was prompted by clinical deterioration, as opposed to elective transfer such as after cardiac surgery. The robustness of these approaches enhances, in our view, the validity of the results.

Our findings may be viewed in light of a paradigm shift in approaches to early detection of the deteriorating patient. This work began in intensive care units. In the neonatal ICU, advanced signal processing and HR time series analysis are used for early detection of neonatal sepsis, where it saves lives[32–34], as well as for other adverse outcomes.[35] In adult ICUs, signatures of illness have been identified in sepsis, respiratory failure leading to urgent intubation and hemorrhage leading to multi-unit transfusion of red blood cells.[36,37] In adult ICU patients already known to be septic, shock may be predicted.[38]

The idea is spreading to the problem of early detection of the deteriorating floor patient. Zimlichman and coworkers have introduced contactless motion detection to derive HR and RR.[39–41] Tarassenko and coworkers developed a probability-based model of patient status from distributions of VS.[42] Hu and coworkers have used advanced data mining to analyze monitor alarms to produce SuperAlarms.[43] Churpek, Edelson and coworkers have devised EMR-based alerts of increasing risk of in-hospital cardiac arrest.[2]

In this work, we introduce advanced time series analysis of continuous ECG telemetric monitoring to capture physiological dynamics in acute care patients. We captured these dynamics in the time, frequency, and non-linear dynamical domains.[14,15,17,18,44] We see many opportunities to apply these and other mathematical approaches to large, well-annotated clinical data sets to develop sophisticated and useful monitoring that use all available data.

### Limitations

This is a single-center observational study in which Cardiovascular Medicine and Cardiothoracic Surgery were the predominant provider teams and thus our results may not generalize to other environments. MET or Rapid Response teams are varied in their composition and implementation across institutions. Because MET activation at our institution is not restricted to objective criteria, we included only MET activations that were followed by ICU transfer. We employed advanced logistic regression models, but note that ensemble methods in machine learning are well-suited for classification of heterogeneous groups.

## Conclusions

Integration of cardiorespiratory dynamics measured from continuous ECG monitoring with intermittent vitals and LABS improves the predictive validity of models to detect clinical deterioration in acute care patients. We look forward to improved care through earlier detection of patient deterioration that incorporates advanced medical analytics of continuous physiologic monitoring.
